# PSMA PET/CT in the Brazilian Unified Healthcare System reduces costs with futile salvage therapies in the management of cases of biochemical recurrence of prostate cancer

**DOI:** 10.1590/0100-3984.2024.0024

**Published:** 2024-08-31

**Authors:** Mateos Bogoni, Juliano Julio Cerci, Evelinda Marramon Trindade, Miguel Morita Fernandes da Silva, Marina Bicalho Silveira, Jônatas Luiz Pereira, Murilo de Almeida Luz, Bernardo Corrêa de Almeida Teixeira

**Affiliations:** 1 Hospital Erasto Gaertner, Curitiba, PR, Brazil.; 2 Quanta – Diagnóstico por Imagem, Curitiba, PR, Brazil.; 3 Hospital das Clínicas da Faculdade de Medicina da Universidade de São Paulo (HC-FMUSP), São Paulo, SP, Brazil.; 4 Hospital de Clínicas da Universidade Federal do Paraná (HC-UFPR), Curitiba, PR, Brazil.; 5 CDTN – Centro de Desenvolvimento da Tecnologia Nuclear, Belo Horizonte, MG, Brazil.

**Keywords:** Positron emission tomography computed tomography, Prostatic neoplasms/diagnostic imaging, Neoplasm recurrence, local/therapy, Tomografia por emissão de pósitrons combinada à tomografia computadorizada, Neoplasias da próstata/diagnóstico por imagem, Recidiva local de neoplasia/terapia

## Abstract

**Objective:**

To compare costs between treatment strategies employed prior to and after prostate-specific membrane antigen positron emission tomography/computed tomography (PSMA PET/CT) via the Brazilian Unified Health Care System and their impact on the therapeutic management of biochemical recurrence of prostate cancer.

**Materials and Methods:**

The referring physicians were surveyed on their treatment intentions (strategies) at two different time points: prior to and after PSMA PET/CT. Cost comparison results are presented as median (IQR) for each of the two strategies. The shift in therapeutic management after PSMA PET/CT was also analyzed.

**Results:**

The study sample included 59 patients (mean age: 65.9 years). The PSMA PET/CT result was considered positive in 38 patients (64.4%) and was found to have an impact on the treatment strategy in for 36 patients (61.0%). Prior to PSMA PET/CT, salvage therapy (i.e., treatment with curative intent) was the intended treatment for most patients, and that was significantly less so after the examination (76.3% vs. 45.8%; *p* < 0.001). Conversely, a strategy involving systemic (i.e., palliative) therapy became more common after PSMA PET/CT (23.7% vs. 54.2%; *p* < 0.001). The after-PSMA PET/CT strategy presented higher overall costs than did the before-PSMA PET/CT strategy, in all scenarios evaluated. In all scenarios, nearly half of this cost difference was related to the cost of the PSMA PET/CT itself, the remainder being related to the new treatment choices that stemmed from knowledge of the PSMA PET/CT findings.

**Conclusion:**

For patients treated within the Brazilian Unified Health Care System, PSMA PET/CT presented higher costs in comparison with conventional imaging methods. Adding PSMA PET/CT to the workflow had an impact on therapeutic management, mainly representing a shift from futile curative treatments to systemic palliative ones. The amount of funds that could potentially be saved by not providing such futile treatments would suffice to evaluate roughly two patients with PSMA PET/CT scans for each futile treatment strategy avoided.

## Abstract (pt)

**Objetivo:**

Comparar custos entre estratégias antes e após o exame de PET/CT-PSMA da perspectiva do Sistema Único de Saúde e seu impacto no manejo terapêutico para pacientes com recidiva bioquímica de câncer de próstata.

**Materiais e Métodos:**

Os médicos solicitantes informaram a intenção terapêutica em dois momentos: antes e após o exame. Os resultados de comparação de custo estão apresentados como medianas de custo (p25; p75). A mudança na intenção terapêutica também foi analisada.

**Resultados:**

O estudo envolveu 59 pacientes (idade média: 65,9 anos). A PET/CT-PSMA foi considerada positiva em 38 dos 59 pacientes (64.4%). O exame impactou a estratégia de tratamento para 36 pacientes (61%). Antes da obtenção das informações da PET/CT-PSMA, a terapia de resgate (i.e., com intenção curativa) era o tratamento sugerido para a maioria dos pacientes, e após o exame, reduziu significativamente (76,3% vs 45,8%; *p* < 0,001). Em contrapartida, a terapia sistêmica (i.e., paliativa) aumentou como intenção de tratamento após o exame (23,7% vs 54,2%; *p* < 0,001). A estratégia “após PET/CT-PSMA” apresentou maiores custos em relação à estratégia “antes da PET/CT-PSMA” nos cenários comparados. Cerca de metade da diferença de custos entre as duas estratégias foi relacionada aos custos do exame propriamente ditos, enquanto a outra metade foi relacionada às novas escolhas de tratamento a partir do exame.

**Conclusão:**

Oferecer a PET/CT-PSMA no Sistema Único de Saúde apresentou maiores custos em relação à estratégia com métodos de imagem convencionais e impactou o manejo terapêutico, pelo favorecimento de tratamentos sistêmicos paliativos no lugar de tratamentos curativos fúteis. A quantidade de recursos que poderiam ser poupados ao evitar tratamentos fúteis seria suficiente para avaliar aproximadamente dois pacientes com exames de PET/CT-PSMA para cada estratégia de tratamento fútil evitada.

## INTRODUCTION

In Brazil, prostate cancer is one of the most common neoplasms among men (second only to nonmelanoma skin cancer). There were an estimated 65,840 new cases of prostate cancer per year during the 2020–2022 triennium, translating to an approximate risk of 62.95 cases per 100,000 men, with approximately 3,560 cases in the state of Paraná alone^(1)^.

After the initial diagnosis and staging by risk group, most patients with prostate cancer will receive treatment with curative intent. In general, this initial treatment is carried out in two main ways: surgery or radiotherapy. Over time, approximately 27–53% of patients treated primarily with curative intent will progress to biochemical recurrence, which corresponds to the detection of residual or recurrent disease through identification of elevated prostate specific antigen (PSA) in serial blood assessments, preceding the appearance of clinical or imaging manifestations of viable disease^(2)^. Variations exist in the medical literature regarding the definition of biochemical recurrence, the one most widely used (including by the Brazilian National Ministry of Health) being that considering two consecutive serum PSA measurements ≥ 0.2 ng/mL for patients who have undergone surgery, or an increase in serum PSA ≥ 2 ng/mL above the nadir for those who have undergone radiotherapy^(3,4)^.

In clinical practice, the detection of biochemical recurrence translates to a high suspicion of viable disease, and a detailed patient assessment through clinical examination and imaging is mandatory, because a second curative approach (salvage therapy) might still be effective in some cases. Therefore, the main challenge in this context is to clarify whether the progressive increase in PSA reflects local, regional, or distant disease.

Gallium-68-prostate-specific membrane antigen positron emission tomography/computed tomography (^68^GaPSMA PET/CT, henceforth referred to as PSMA PET/CT) is an imaging method developed in the last decade and based on the intravenous administration of a radiotracer that binds to PSMA molecules, which are highly expressed on the surface of prostate tumor cells^(5,6,7,8)^. In a single hybrid PET/CT scanner, two sets of images are acquired for each patient: PET images, which illustrate how the radiotracer was absorbed throughout the body; and CT images, which provide highly detailed anatomical information.

Several clinical trials have established the high sensitivity and specificity of PSMA PET/CT, especially in the setting of biochemical recurrence^(9,10,11,12,13,14,15,16)^, which led to its adoption as the imaging method of choice in the main international protocols and guidelines, such as those issued by the European Association of Nuclear Medicine, the Society of Nuclear Medicine and Molecular Imaging, the European Association of Urology, and the National Comprehensive Cancer Network^(17,18,19)^. However, the financial and technological realities in most low- and middle-income countries, including Brazil, do not yet allow this recommendation to be strictly followed, because of the high cost and low availability of PSMA PET/CT in comparison with other methods. For the technology to be incorporated into the Brazilian Unified Health Care System (*Sistema Único de Saúde* – SUS), studies of its economic impact are necessary to define the relationship between technology-related expenses and their actual impact on health^(20)^.

The primary objective of the present study was to perform a cost-comparison analysis between pre- and post-PSMA PET/CT treatment strategies in the workup of patients with biochemical recurrence of prostate cancer treated via the SUS. We attempted to determine whether there were any differences in costs between the two strategies and which factors promote such differences. As a secondary objective, we assessed the impact that PSMA PET/CT had on therapeutic management. This might provide valuable information for the development of cost-effective analyses in the future.

## MATERIALS AND METHODS

### Patients

The patients in our study sample were selected from the cohort evaluated in a recently published international multicenter study promoted by the International Atomic Energy Agency (IAEA), which enrolled over a thousand patients worldwide^(21)^. The goal of that study was to evaluate the use of PSMA PET/CT in the setting of biochemical recurrence of prostate cancer in 15 countries around the globe. Of the 165 patients in the Brazilian cohort, 59 were treated via the SUS and were therefore selected for inclusion in the present study. All 59 of those patients were evaluated and followed at Hospital Erasto Gaertner, in the city of Curitiba, Brazil, and all underwent PSMA PET/CT at the same private diagnostic imaging clinic. All of the patients met the criteria for biochemical recurrence after primary treatment with curative intent, were submitted to PSMA PET/CT, and were followed for at least 6 months.

The inclusion criteria were as follows: age ≥ 18 years; histologically proven prostate adenocarcinoma; previous treatment with curative intent (radical prostatectomy or radiotherapy); and biochemical recurrence defined as an increase in serum PSA (to ≥ 0.2 ng/mL) confirmed in two consecutive measurements (after radical prostatectomy), or as an absolute increase in serum PSA of ≥ 2 ng/mL above the nadir (after radiotherapy). Patients with a PSA of 4–10 ng/mL were considered eligible only if conventional imaging methods (CT and bone scintigraphy) were negative. Patients with a history of another type of cancer were excluded, as were those with a history of Paget’s disease and those with a PSA ≥ 10 ng/mL.

The committee responsible for the multicenter study designed a standardized data collection form, which was completed for each patient. These forms were filled out in a multidisciplinary manner, by the team of attending physicians (urologists and oncologists) and the imaging team (radiologists and nuclear physicians), later being reviewed by the principal investigators from each center prior to submission to the IAEA. All participating centers obtained approval from the respective local ethics committees, and all participating patients gave written informed consent.

### Data

**Figure 1:**
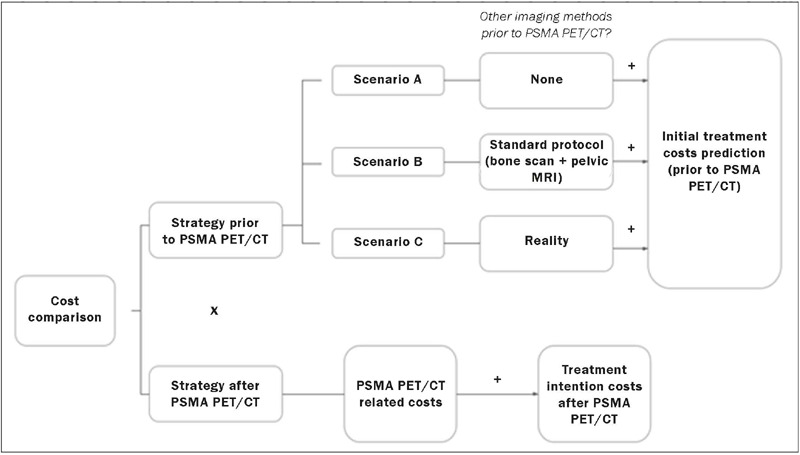
Flow chart of the cost-comparison study.

Because the forms obtained from the multicenter IAEA study contained multiple data for each patient, we must highlight what was ultimately used in our analysis. The objective was to compare costs between two strategies (Figure 1): that employed prior to PSMA PET/CT; and that employed after. In order to facilitate peer review, all costs are presented in U.S. dollars.

The first strategy (the before-PSMA PET/CT strategy) was associated with the following costs:

*a) The costs of other imaging prior to PSMA PET/C T.* These are the costs related to traditional imaging methods that may have been performed: bone scintigraphy ($39.54), pelvic magnetic resonance imaging (MRI, $55.64), abdominal CT ($28.65), and transrectal ultrasound ($19.12). This information was handled differently in three distinct scenarios, which will be further explained.
*b) Predicted costs of initially intended treatment.* These are the costs related to therapies to which the patients would have been submitted if the PSMA PET/CT findings had not been provided. Information regarding the intended treatment prior to PSMA PET/CT was requested on the standard data collection forms.

The second strategy (the after-PSMA PET/CT strategy) was associated with the following costs:

*a) Costs directly related to PSMA PET/CT.* These costs include radiotracer production, machinery, personnel, etc.
*b) Costs of the intended treatment after PSMA PET/CT.* These are the costs related to the new therapies to which the patients will be submitted at the behest of the team of attending physicians after the PSMA PET/CT findings are known.

Except for the PSMA PET/CT costs (which were obtained by consulting the financial sector of the private imaging clinic at which the scans were performed, estimated to be $524.19), all other costs were obtained from the perspective of the public health care system as payer, by accessing the SUS reimbursement list on the *Sistema de Gerenciamento da Tabela de Procedimentos, Medicamentos, Órteses, Próteses e Materiais Especiais do Departamento de Informática do SUS* (SIGTAP-DATASUS, System for the Management of the Table of Procedures, Medications, Orthoses, Prostheses, and Special Materials of the Information Technology Department of the SUS) platform. The SIGTAP-DATASUS is an online, open-access Brazilian government system that lists the official fees and conditions for all procedures and medications offered by the SUS^(22)^. The costs were estimated considering a 24-month period, with guidance from the team of attending physicians about which costs to consider.

For both strategies, the treatment intentions were divided in two broad groups: salvage and systemic (i.e., curative and palliative, respectively), with four salvage therapy options—radical prostatectomy ($134.06), pelvic lymphadenectomy ($901.84), exclusive radiotherapy ($1,224.41), and combined radiotherapy and androgen deprivation therapy ($4,871.79)—and four systemic therapy options—androgen deprivation therapy ($3,647.38), chemotherapy ($160.62), bilateral orchiectomy ($105.04), and active surveillance ($157.30). All of the costs related to the intended treatments and imaging examinations are detailed in Tables 1 and 2.

**Table 1:** Costs related to the intended treatments and imaging examinations.

Costs of the intended treatments (24-month period)																		
RP	Initial outpatient appointment: $2.07		Initial PSA measurement: $3.40			Laparoscopic RP: $123.12			Inpatient visitation round: $0			Second outpatient appointment: $2.07				Second PSA measurement: $3.40		= $134.06
PL	Initial outpatient appointment: $2.07		Initial PSA measurement: $3.40			PL: $890.90			Inpatient visitation round: $0			Second outpatient appointment: $2.07				Second PSA measurement: $3.40		= $901.84
RT	Initial outpatient appointment: $2.07		Initial PSA measurement: $3.40			Hospital admission for RT: $4.78			Prostate RT: $1,208.70			Second outpatient appointment: $2.07				Second PSA measurement: $3.40		= $1,224.41
ADT	Outpatient appointment every 3 months (8 total): $16.56			Zoladex or Eligard every 3 months (8 total): $3,586.34					PSA measurement every 3 months (8 total): $27.20					Testosterone measurement every 3 months (8 total): $17.28				= $3,647.38
RT + ADT	RT costs: $1,224.41								ADT costs: $3,647.38									= $4,871.79
ChT	Initial outpatient appointment: $2.07		Initial PSA measurement: $3.40		Docetaxel every 3 weeks (6 cycles): $148.93				Prednisone every 12 h for 5 days (6 cycles): $0.75				Second outpatient appointment: $2.07				Second PSA measurement: $3.40	= $160,62
ORC	Initial outpatient appointment: $2.07	Initial PSA measurement $3.40		Initial testosterone : measurement: $2.16			ORC: $89.78		Inpatient visitation round: $0	Outpatient appointment: $2.07			Second PSA measurement: $3.40			Second testosterone measurement: $2.16		= $105,04
SRV	Outpatient appointment every 6 months (4 total): $8.28			PSA measurement every 6 months (4 total): $13.60				Superior abdomen MRI: $55.64			Pelvic MRI: $55.64				Ultrasound-guided transrectal prostate biopsy: $24.14			= $157,30
Imaging costs*																		
^68^Ga-PSMA PET/CT: $524.19			Pelvic MRI: $55.64			Bone scintigraphy: $39.54						Abdominal CT: $28.65					Transrectal ultrasound: $19.12	

RP, radical prostatectomy; PL, pelvic lymphadenectomy; RT, radiation therapy; ADT, androgen deprivation therapy; Zoladex, goserelin acetate; Eligard, leuprolide acetate; ChT, chemotherapy; ORC, (bilateral) orchiectomy; SRV, (active) surveillance.

*^68^Ga-PSMA PET/CT costs were obtained by consulting the financial sector of the private imaging facility at which the scans were performed. For detailed information on the calculated costs, please refer to Table 2; all other imaging costs were obtained by consulting public data available on the SIGTAP-DATASUS website of the Brazilian National Ministry of Health.

**Table 2:** PSMA PET/CT-related costs.

Item(s)	Cost in U.S. dollars	Cost in Brazilian reals
Nursing supplies*	$10.88	R$52.56
Machinery†	$123.47	R$596.36
On-site ^68^Ga-PSMA synthesis	$389.84	R$1,882.90
Total	$524.19	R$2,531.84

* Nursing supplies include items such as syringes, needles, bandages, saline solution, medication (furosemide), iodinated contrast, and gloves.

† Machinery costs include those related to purchase financing and general maintenance.

### Cost-comparison analysis

The conversion from the original currency (Brazilian reals) was made on the basis of the mean exchange rate over the three-year study period (2019–2021), resulting in 1 U.S. dollar being equal to 4.83 Brazilian reals, according to official data from the Brazilian government^(23)^. The costs prior to and after PSMA PET/CT were tabulated for each individual patient (Tables 3 and 4, respectively).

**Table 3:** Costs prior to PSMA PET/CT tabulated for each patient.

Patient	Intended treatment prior to PSMA PET/CT	Predicted initial treatment cost	Scenario A			Scenario B			Scenario C		
			Initial imaging	Imaging costs	Total costs	Initial imaging	Imaging costs	Total costs	Initial imaging	Imaging costs	Total costs
1	ADT	$3,647.59	None	$0.00	$3,647.59	Pelvic MRI + BS	$95.18	$3,742.77	Pelvic MRI + CT + BS	$123.83	$3,771.42
2	RT	$1,224.41	None	$0.00	$1,224.41	Pelvic MRI + BS	$95.18	$1,319.60	N/A	$0.00	$1,224.41
3	RT	$1,224.41	None	$0.00	$1,224.41	Pelvic MRI + BS	$95.18	$1,319.60	BS	$39.54	$1,263.96
4	RT + ADT	$4,871.79	None	$0.00	$4,871.79	Pelvic MRI + BS	$95.18	$4,966.98	Pelvic MRI + CT + BS	$123.83	$4,995.62
5	ADT	$3,647.59	None	$0.00	$3,647.59	Pelvic MRI + BS	$95.18	$3,742.77	Pelvic MRI + CT	$84.29	$3,731.87
6	ADT	$3,647.59	None	$0.00	$3,647.59	Pelvic MRI + BS	$95.18	$3,742.77	Pelvic MRI + CT + BS	$123.83	$3,771.42
7	RT	$1,224.41	None	$0.00	$1,224.41	Pelvic MRI + BS	$95.18	$1,319.60	N/A	$0.00	$1,224.41
8	RT	$1,224.41	None	$0.00	$1,224.41	Pelvic MRI + BS	$95.18	$1,319.60	Pelvic MRI	$55.64	$1,280.06
9	RP	$134.06	None	$0.00	$134.06	Pelvic MRI + BS	$95.18	$229.25	Pelvic MRI + CT + BS	$123.83	$257.89
10	RT	$1,224.41	None	$0.00	$1,224.41	Pelvic MRI + BS	$95.18	$1,319.60	CT + BS	$68.19	$1,292.60
11	RT	$1,224.41	None	$0.00	$1,224.41	Pelvic MRI + BS	$95.18	$1,319.60	Pelvic MRI	$55.64	$1,280.06
12	RT	$1,224.41	None	$0.00	$1,224.41	Pelvic MRI + BS	$95.18	$1,319.60	N/A	$0.00	$1,224.41
13	PL	$901.84	None	$0.00	$901.84	Pelvic MRI + BS	$95.18	$997.02	CT + BS	$68.19	$970.03
14	RT	$1,224.41	None	$0.00	$1,224.41	Pelvic MRI + BS	$95.18	$1,319.60	Pelvic MRI + CT + BS	$123.83	$1,348.24
15	ADT	$3,647.59	None	$0.00	$3,647.59	Pelvic MRI + BS	$95.18	$3,742.77	CT + BS	$68.19	$3,715.77
16	RT	$1,224.41	None	$0.00	$1,224.41	Pelvic MRI + BS	$95.18	$1,319.60	Pelvic MRI + CT + BS	$123.83	$1,348.24
17	PL	$901.84	None	$0.00	$901.84	Pelvic MRI + BS	$95.18	$997.02	Pelvic MRI + CT + BS	$123.83	$1,025.67
18	RP	$134.06	None	$0.00	$134.06	Pelvic MRI + BS	$95.18	$229.25	Pelvic MRI + CT + TRUS	$103.41	$237.48
19	RT + ADT	$4,871.79	None	$0.00	$4,871.79	Pelvic MRI + BS	$95.18	$4,966.98	Pelvic MRI	$55.64	$4,927.43
20	RT	$1,224.41	None	$0.00	$1,224.41	Pelvic MRI + BS	$95.18	$1,319.60	Pelvic MRI + CT + BS	$123.83	$1,348.24
21	ADT	$3,647.59	None	$0.00	$3,647.59	Pelvic MRI + BS	$95.18	$3,742.77	CT + BS	$68.19	$3,715.77
22	RT	$1,224.41	None	$0.00	$1,224.41	Pelvic MRI + BS	$95.18	$1,319.60	Pelvic MRI + CT + BS	$123.83	$1,348.24
23	ADT	$3,647.59	None	$0.00	$3,647.59	Pelvic MRI + BS	$95.18	$3,742.77	Pelvic MRI + CT + BS	$123.83	$3,771.42
24	RT	$1,224.41	None	$0.00	$1,224.41	Pelvic MRI + BS	$95.18	$1,319.60	Pelvic MRI + CT + BS	$123.83	$1,348.24
25	ADT	$3,647.59	None	$0.00	$3,647.59	Pelvic MRI + BS	$95.18	$3,742.77	Pelvic MRI + CT	$84.29	$3,731.87
26	ADT	$3,647.59	None	$0.00	$3,647.59	Pelvic MRI + BS	$95.18	$3,742.77	Pelvic MRI	$55.64	$3,703.23
27	RT+ADT	$4,871.79	None	$0.00	$4,871.79	Pelvic MRI + BS	$95.18	$4,966.98	Pelvic MRI + CT + BS	$123.83	$4,995.62
28	RT	$1,224.41	None	$0.00	$1,224.41	Pelvic MRI + BS	$95.18	$1,319.60	N/A	$0.00	$1,224.41
29	ADT	$3,647.59	None	$0.00	$3,647.59	Pelvic MRI + BS	$95.18	$3,742.77	Pelvic MRI + CT + BS	$123.83	$3,771.42
30	RT + ADT	$4,871.79	None	$0.00	$4,871.79	Pelvic MRI + BS	$95.18	$4,966.98	N/A	$0.00	$4,871.79
31	RT + ADT	$4,871.79	None	$0.00	$4,871.79	Pelvic MRI + BS	$95.18	$4,966.98	Pelvic MRI + CT + BS	$123.83	$4,995.62
32	ChT	$160.62	None	$0.00	$160.62	Pelvic MRI + BS	$95.18	$255.80	CT	$28.65	$189.26
33	RT	$1,224.41	None	$0.00	$1,224.41	Pelvic MRI + BS	$95.18	$1,319.60	N/A	$0.00	$1,224.41
34	RT + ADT	$4,871.79	None	$0.00	$4,871.79	Pelvic MRI + BS	$95.18	$4,966.98	BS	$39.54	$4,911.34
35	RT	$1,224.41	None	$0.00	$1,224.41	Pelvic MRI + BS	$95.18	$1,319.60	Pelvic MRI	$55.64	$1,280.06
36	RT + ADT	$4,871.79	None	$0.00	$4,871.79	Pelvic MRI + BS	$95.18	$4,966.98	Pelvic MRI + CT + BS	$123.83	$4,995.62
37	RT	$1,224.41	None	$0.00	$1,224.41	Pelvic MRI + BS	$95.18	$1,319.60	Pelvic MRI + CT + BS	$123.83	$1,348.24
38	RT	$1,224.41	None	$0.00	$1,224.41	Pelvic MRI + BS	$95.18	$1,319.60	Pelvic MRI + CT + BS	$123.83	$1,348.24
39	RT	$1,224.41	None	$0.00	$1,224.41	Pelvic MRI + BS	$95.18	$1,319.60	N/A	$0.00	$1,224.41
40	ORC	$105.04	None	$0.00	$105.04	Pelvic MRI + BS	$95.18	$200.22	BS	$39.54	$144.58
41	PL	$901.84	None	$0.00	$901.84	Pelvic MRI + BS	$95.18	$997.02	Pelvic MRI + CT + BS	$123.83	$1,025.67
42	RT + ADT	$4,871.79	None	$0.00	$4,871.79	Pelvic MRI + BS	$95.18	$4,966.98	Pelvic MRI + CT + BS	$123.83	$4,995.62
43	RT	$1,224.41	None	$0.00	$1,224.41	Pelvic MRI + BS	$95.18	$1,319.60	N/A	$0.00	$1,224.41
44	RT	$1,224.41	None	$0.00	$1,224.41	Pelvic MRI + BS	$95.18	$1,319.60	Pelvic MRI	$55.64	$1,280.06
45	RT	$1,224.41	None	$0.00	$1,224.41	Pelvic MRI + BS	$95.18	$1,319.60	Pelvic MRI + BS	$123.83	$1,348.24
46	RT	$1,224.41	None	$0.00	$1,224.41	Pelvic MRI + BS	$95.18	$1,319.60	CT + BS	$68.19	$1,292.60
47	RT	$1,224.41	None	$0.00	$1,224.41	Pelvic MRI + BS	$95.18	$1,319.60	Pelvic MRI + CT + BS	$123.83	$1,348.24
48	RT	$1,224.41	None	$0.00	$1,224.41	Pelvic MRI + BS	$95.18	$1,319.60	CT + BS	$68.19	$1,292.60
49	RT + ADT	$4,871.79	None	$0.00	$4,871.79	Pelvic MRI + BS	$95.18	$4,966.98	Pelvic MRI + CT + BS	$123.83	$4,995.62
50	ADT	$3,647.59	None	$0.00	$3,647.59	Pelvic MRI + BS	$95.18	$3,742.77	Pelvic MRI + CT + BS	$123.83	$3,771.42
51	RT	$1,224.41	None	$0.00	$1,224.41	Pelvic MRI + BS	$95.18	$1,319.60	BS	$39.54	$1,263.96
52	RT	$1,224.41	None	$0.00	$1,224.41	Pelvic MRI + BS	$95.18	$1,319.60	N/A	$0.00	$1,224.41
53	ADT	$3,647.59	None	$0.00	$3,647.59	Pelvic MRI + BS	$95.18	$3,742.77	Pelvic MRI + CT + BS	$123.83	$3,771.42
54	RT	$1,224.41	None	$0.00	$1,224.41	Pelvic MRI + BS	$95.18	$1,319.60	Pelvic MRI + CT + BS	$123.83	$1,348.24
55	RT	$1,224.41	None	$0.00	$1,224.41	Pelvic MRI + BS	$95.18	$1,319.60	Pelvic MRI + CT + BS	$123.83	$1,348.24
56	RT + ADT	$4,871.79	None	$0.00	$4,871.79	Pelvic MRI + BS	$95.18	$4,966.98	Pelvic MRI + CT + BS	$123.83	$4,995.62
57	RT + ADT	$4,871.79	None	$0.00	$4,871.79	Pelvic MRI + BS	$95.18	$4,966.98	Pelvic MRI + CT + BS	$123.83	$4,995.62
58	RT	$1,224.41	None	$0.00	$1,224.41	Pelvic MRI + BS	$95.18	$1,319.60	BS	$39.54	$1,263.96
59	ADT	$3,647.59	None	$0.00	$3,647.59	Pelvic MRI + BS	$95.18	$3,742.77	Pelvic MRI + CT + BS	$123.83	$3,771.42

ADT, androgen deprivation therapy; RT, radiation therapy; RP, radical prostatectomy; PL, pelvic lymphadenectomy; ChT, chemotherapy; BS, bone scintigraphy; TRUS, transrectal ultrasound; ORC, (bilateral) orchiectomy.

**Table 4:** Costs after PSMA PET/CT tabulated for each patient.

Patient	Intended treatment after PSMA PET/CT	Predicted cost of treatment after PSMA PET/CT	PSMA PET/CT imaging costs	Total costs after PSMA PET/CT (treatment + imaging)	Cost difference between before- and after-PSMA PET/CT treatment strategies		
					Scenario A	Scenario B	Scenario C
1	RT + ADT	$4,871.79	$524.19	$5,395.98	+$1,748.39	+$1,653.21	+$1,624.56
2	RT + ADT	$4,871.79	$524.19	$5,395.98	+$4,171.57	+$4,076.38	+$4,171.57
3	RT + ADT	$4,871.79	$524.19	$5,395.98	+$4,171.57	+$4,076.38	+$4,132.02
4	SRV	$157.30	$524.19	$681.49	−$4,190.31	−$4,285.49	−$4,314.14
5	ADT	$3,647.59	$524.19	$4,171.77	+$524.19	+$429.00	+$439.90
6	ORC	$105.04	$524.19	$629.22	−$3,018.36	−$3,113.55	−$3,142.19
7	RT + ADT	$4,871.79	$524.19	$5,395.98	+$4,171.57	+$4,076.38	+$4,171.57
8	RT + ADT	$4,871.79	$524.19	$5,395.98	+$4,171.57	+$4,076.38	+$4,115.92
9	ADT	$3,647.59	$524.19	$4,171.77	+$4,037.71	+$3,942.53	+$3,913.88
10	SRV	$157.30	$524.19	$681.49	−$542.93	−$638.11	−$611.12
11	SRV	$157.30	$524.19	$681.49	−$542.93	−$638.11	−$598.57
12	ADT	$3,647.59	$524.19	$4,171.77	+$2,947.36	+$2,852.17	+$2,947.36
13	ADT	$3,647.59	$524.19	$4,171.77	+$3,269.93	+$3,174.75	+$3,201.74
14	RT	$1,224.41	$524.19	$1,748.60	+$524.19	+$429.00	+$400.36
15	ADT	$3,647.59	$524.19	$4,171.77	+$524.19	+$429.00	+$456.00
16	ADT	$3,647.59	$524.19	$4,171.77	+$2,947.36	+$2,852.17	+$2,823.53
17	ORC	$105.04	$524.19	$629.22	−$272.62	−$367.80	−$396.45
18	RT + ADT	$4,871.79	$524.19	$5,395.98	+$5,261.92	+$5,166.73	+$5,158.50
19	RT + ADT	$4,871.79	$524.19	$5,395.98	+$524.19	+$429.00	+$468.54
20	ChT	$160.62	$524.19	$684.80	−$539.61	−$634.80	−$663.44
21	ADT	$3,647.59	$524.19	$4,171.77	+$524.19	+$429.00	+$456.00
22	ChT	$160.62	$524.19	$684.80	−$539.61	−$634.80	−$663.44
23	ADT	$3,647.59	$524.19	$4,171.77	+$524.19	+$429.00	+$400.36
24	ADT	$3,647.59	$524.19	$4,171.77	+$2,947.36	+$2,852.17	+$2,823.53
25	ADT	$3,647.59	$524.19	$4,171.77	+$524.19	+$429.00	+$439.90
26	RT + ADT	$4,871.79	$524.19	$5,395.98	+$1,748.39	+$1,653.21	+$1,692.75
27	ADT	$3,647.59	$524.19	$4,171.77	−$700.02	−$795.20	−$823.85
28	ADT	$3,647.59	$524.19	$4,171.77	+$2,947.36	+$2,852.17	+$2,947.36
29	ADT	$3,647.59	$524.19	$4,171.77	+$524.19	+$429.00	+$400.36
30	RT + ADT	$4,871.79	$524.19	$5,395.98	+$524.19	+$429.00	+$524.19
31	RT + ADT	$4,871.79	$524.19	$5,395.98	+$524.19	+$429.00	+$400.36
32	ADT	$3,647.59	$524.19	$4,171.77	+$4,011.16	+$3,915.97	+$3,982.51
33	ADT	$3,647.59	$524.19	$4,171.77	+$2,947.36	+$2,852.17	+$2,947.36
34	ADT	$3,647.59	$524.19	$4,171.77	−$700.02	−$795.20	−$739.56
35	SRV	$157.30	$524.19	$681.49	−$542.93	−$638.11	−$598.57
36	ADT	$3,647.59	$524.19	$4,171.77	−$700.02	−$795.20	−$823.85
37	SRV	$157.30	$524.19	$681.49	−$542.93	−$638.11	−$666.76
38	RT	$1,224.41	$524.19	$1,748.60	+$524.19	+$429.00	+$400.36
39	ADT	$3,647.59	$524.19	$4,171.77	+$2,947.36	+$2,852.17	+$2,947.36
40	ADT	$3,647.59	$524.19	$4,171.77	+$4,066.74	+$3,971.55	+$4,027.19
41	ADT	$3,647.59	$524.19	$4,171.77	+$3,269.93	+$3,174.75	+$3,146.10
42	RT + ADT	$4,871.79	$524.19	$5,395.98	+$524.19	+$429.00	+$400.36
43	RT	$1,224.41	$524.19	$1,748.60	+$524.19	+$429.00	+$1,748.60
44	RT	$1,224.41	$524.19	$1,748.60	+$524.19	+$429.00	+$468.54
45	RT	$1,224.41	$524.19	$1,748.60	+$524.19	+$429.00	+$400.36
46	RT	$1,224.41	$524.19	$1,748.60	+$524.19	+$429.00	+$456.00
47	RT	$1,224.41	$524.19	$1,748.60	+$524.19	+$429.00	+$400.36
48	RT	$1,224.41	$524.19	$1,748.60	+$524.19	+$429.00	+$456.00
49	RT + ADT	$4,871.79	$524.19	$5,395.98	+$524.19	+$429.00	+$400.36
50	ADT	$3,647.59	$524.19	$4,171.77	+$524.19	+$429.00	+$400.36
51	RT	$1,224.41	$524.19	$1,748.60	+$524.19	+$429.00	+$484.64
52	RT	$1,224.41	$524.19	$1,748.60	+$524.19	+$429.00	+$524.19
53	ADT	$3,647.59	$524.19	$4,171.77	+$524.19	+$429.00	+$400.36
54	RT	$1,224.41	$524.19	$1,748.60	+$524.19	+$429.00	+$400.36
55	RT	$1,224.41	$524.19	$1,748.60	+$524.19	+$429.00	+$1,748.60
56	RT + ADT	$4,871.79	$524.19	$5,395.98	+$524.19	+$429.00	+$400.36
57	RT + ADT	$4,871.79	$524.19	$5,395.98	+$524.19	+$429.00	+$400.36
58	RT	$1,224.41	$524.19	$1,748.60	+$524.19	+$429.00	+$1,748.60
59	ADT	$3,647.59	$524.19	$4,171.77	+$524.19	+$429.00	+$400.36

RP, radical prostatectomy; RT, radiation therapy; ADT, androgen deprivation therapy; ChT, chemotherapy; ORC, (bilateral) orchiectomy; SRV, (active) surveillance.

In the before-PSMA PET/CT strategy, three distinct cost scenarios (designated A, B, and C) were created and compared. The difference among the three scenarios is regarding the costs related to imaging methods that might be performed prior to PSMA PET/CT. In scenario A, no imaging costs prior to PSMA PET/CT were taken into account, as if PSMA PET/CT had been the first imaging study performed. In scenario B, all patients were assumed to have been submitted to bone scintigraphy and pelvic MRI, which is the standard protocol in cases of biochemical recurrence at the institution. In scenario C, the costs reflected the real imaging methods to which each patient was submitted, which turned out to be quite heterogeneous. The reasoning behind creating these three scenarios was to account for the possible cost impact of imaging methods other than PSMA PET/CT if they were not performed at all (scenario A), if all patients were submitted to the standard protocol (scenario B), and if the reality in our sample is considered (scenario C). For both strategies (prior to and after PSMA PET/CT), all other costs (i.e., those related to the initial intended treatment prior to PSMA PET/CT, those related to the intended treatment after PSMA PET/CT, and those related to PSMA PET/CT imaging) were preserved in the three scenarios.

### Statistical analysis

In each scenario, the cost differences (between the before- and after-PSMA PET/CT strategies) were calculated for each patient. The Shapiro-Wilk test rejected normal distribution for those cost differences, and the sign test was therefore chosen for statistical analysis. For each of the three scenarios, the results are presented as median (IQR) for each of the two strategies (prior to and after PSMA PET/CT). The impact of PSMA PET/CT on the treatment strategy was considered positive when there was a change in the strategy after PSMA PET/CT. The shift in therapeutic management between the salvage and systemic categories after PSMA PET/CT was analyzed by using McNemar’s test. All statistical analyses were performed with the Stata statistical software package, version 15.0 (StataCorp, College Station, TX, USA), and the level of significance was defined as *p* < 0.05.

## RESULTS

The mean age of the patients was 65.9 ± 7.7 years. The PSMA PET/CT was considered positive (i.e., was able to identify at least one suspected site of disease with PSMA expression) in 38 (64.4%) of the 59 patients. This was similar to the 65.1% positivity rate reported in the IAEA multicenter study^(21)^.

Overall, 44 patients (74.6%) had been submitted to prostatectomy as the primary treatment and 15 (25.4%) had been submitted to radiotherapy. The Gleason score (GS) at prostate biopsy presented the following distribution: GS7, in 39 patients (66.1%); GS8, in 10 (16.9%); GS9, in nine (15.2%); and GS10, in one (1.7%). The median PSA level at the time of PSMA PET/CT (from two weeks before up to two weeks after the scan) was 1.7 ng/mL (IQR, 0.4–8.2). The mean PSA doubling time was 11.2 ± 8.9 months. These patients were followed for a mean of 13.4 months (range, 6.0–29.0 months).

**Figure 2:**
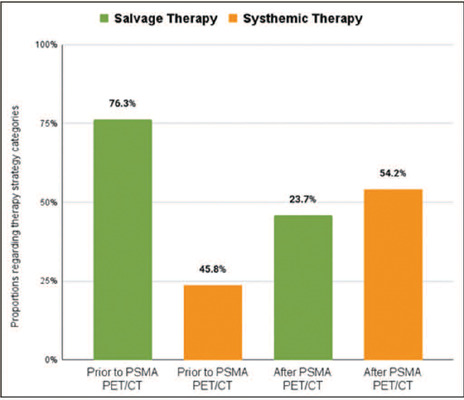
Proportions of patients by treatment strategy prior to and after PSMA PE T/CT.

**Figure 3:**
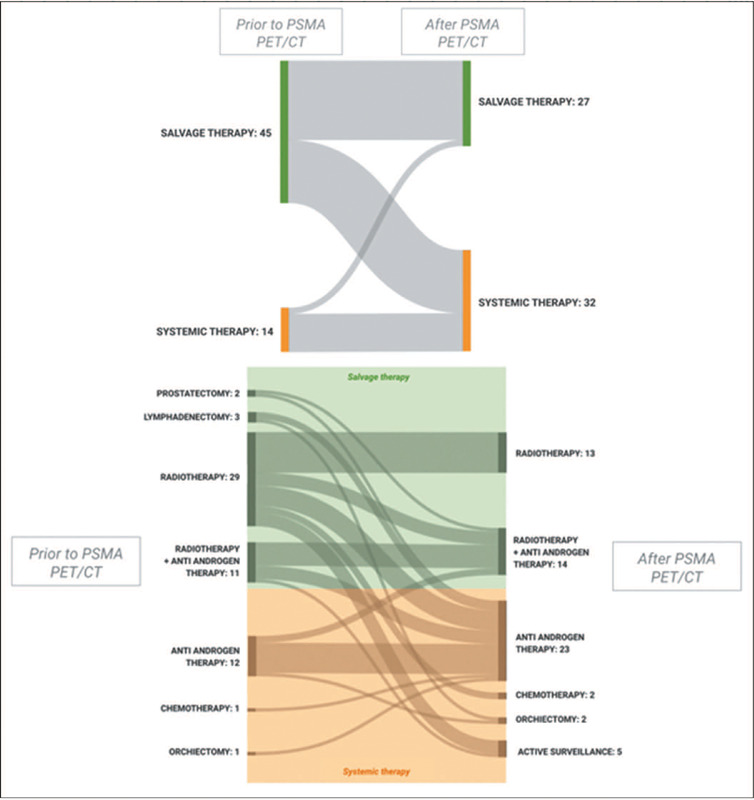
Sankey diagrams illustrating the impact of PSMA PET/CT on therapeutic management by category (salvage and systemic) and by time point (prior to and after PSMA PET/CT).

**Figure 4:**
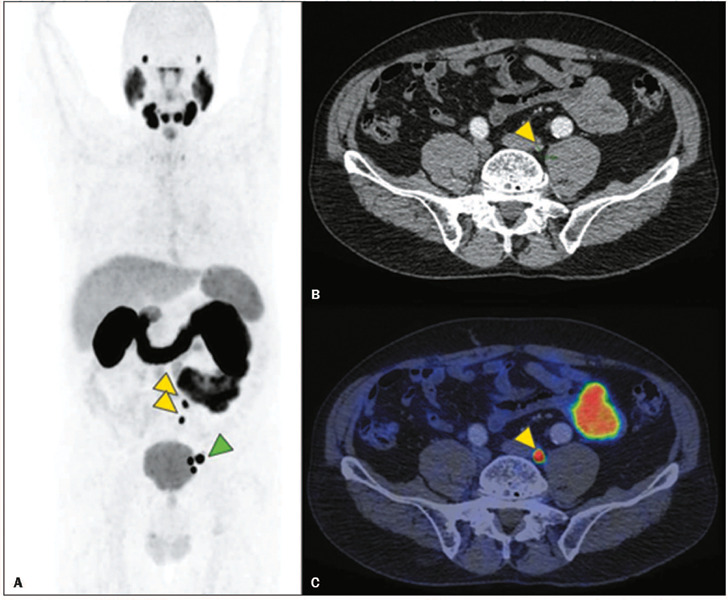
Example of PSMA PET/CT findings that have an impact on therapeutic management. **A:** Three-dimensional maximum intensity projection PET reconstruction. **B:** CT. **C:** PET/CT fusion images. Although CT is able to show metastases in left internal iliac lymph nodes because of their large dimensions (green arrowhead), based solely on this finding, this patient would be classified as N1M0 (regional lymph nodes involvement without distant metastases) and hence a possible candidate for salvage therapy (with curative intent). However, PSMA PET/CT also showed involvement of left common iliac lymph nodes, which was possible only because of high PSMA expression (yellow arrowheads in **A** and **C**), given that the size and contours of these lymph nodes are preserved (yellow arrowhead in **B**), which led them to be considered normal by CT criteria. Thus, this patient was correctly classified by PSMA PET/CT as N1M1 (since common iliac lymph nodes involvement is not considered regional), and the therapeutic management was altered to a systemic (palliative) strategy.

An effective impact of PSMA PET/CT on the treatment strategy was observed in 36 (61.0%) of the 59 patients, which is comparable to the 56.8% observed in the IAEA study^(21)^. A priori salvage therapy was the intended treatment for 45 patients (76.3%), through radiotherapy in 29, through combined radiotherapy and androgen deprivation therapy in 11, through lymphadenectomy in three, and through prostatectomy in two. Systemic therapy was the intended treatment for the other 14 patients (23.7%). After the PSMA PET/CT results were known, this scale was shifted. Systemic therapy became the most prevalent intended treatment, being chosen for 32 patients (54.2%), compared with 27 patients (45.8%) for whom salvage therapy was chosen (Figures 2 and 3). Of those 27 patients, none had surgery (prostatectomy or lymphadenectomy) indicated as the new intended treatment, whereas five had been scheduled to undergo surgery with curative intent before PSMA PET/CT (lymphadenectomy in three and prostatectomy in two). Table 5, Figure 2, and Figure 3 illustrate the distribution of the intended treatments prior to and after PSMA PET/CT. Figure 4 illustrates the type of PSMA PET/CT finding that can lead to a change in the treatment strategy.

**Table 5:** Treatment strategies prior to and after PSMA PET/CT (N = 59).

Intended treatment	Prior to PSMA PET/CT n (%)	After PSMA PET/CT n (%)	*P*
RP (salvage therapy)	2 (3.4)	0	0.15
PL (salvage therapy)	3 (5.1)	0	0.08
RT alone (salvage therapy)	29 (49.1)	13 (22.0)	< 0.001
RT + ADT (salvage therapy)	11 (18.6)	14 (23.7)	0.08
ADT (systemic therapy)	12 (20.3)	23 (39.0)	< 0.05
ChT (systemic therapy)	1 (1.7)	2 (3.4)	0.3
ORC (systemic therapy)	1 (1.7)	2 (3.4)	0.3
SRV (systemic therapy)	0	5 (8.5)	< 0.05

RP, radical prostatectomy; PL, pelvic lymphadenectomy; RT, radiation therapy; ADT, androgen deprivation therapy; ChT, chemotherapy; ORC, (bilateral) orchiectomy; SRV, (active) surveillance.

Following our analysis of the impact of PSMA PET/CT on treatment intentions, we proceeded to evaluate the results regarding the costs involved. In all three scenarios, the after-PSMA PET/CT strategy presented higher overall costs than did the before-PSMA PET/CT strategy (Table 6), with the cost difference between the two strategies being highest ($1,087.35) for scenario A and lowest ($992.17) for scenario B, which translates to a mean difference of $95.18 between the scenarios (as expected, representing the exact cost of performing the standard protocol of bone scintigraphy + pelvic MRI prior to PSMA PET/CT per patient, which is the actual distinguishing factor between those two scenarios).

**Table 6:** Costs prior to and after PSMA PET/CT for each scenario.

Scenario*	Prior to PSMA PET/CT	After PSMA PET/CT	Difference	P
A				
Mean Median (IQR)	$230.69 $122.44 (122.44–364.76)	$339.43 $ 417.1 8 (174.86–417.18)	$1,087.35	< 0.001
B				
Mean Median (IQR)	$240.21 $131.96 (131.96–374.28)	$339.43 $ 417.1 8 (174.86–417.18)	$992.17	< 0.001
C				
Mean Median (IQR)	$238.88 $134.82 (122.44–170.10)	$339.43 $ 417.1 8 (174.86–417.18)	$1,005.45	< 0.001

* For the before-PSMA PET/CT strategy, three distinct cost scenarios were created and compared. The difference between the three scenarios was regarding the costs related to eventual imaging methods performed prior to PSMA PET/CT. In scenario A, no imaging method costs prior to PSMA PET/CT were taken into account, as if PSMA PET/CT had been the first imaging study performed. In scenario B, all patients were assumed to have been submitted to bone scintigraphy + pelvic MRI, which is the standard protocol in cases of biochemical recurrence. In scenario C, the costs reflected the actual imaging methods to which each patient was submitted.

## DISCUSSION

In the management of prostate cancer, PSMA PET/CT is an established imaging method. Over time, the indications for PSMA PET/CT have grown^(24,25,26)^: first in biochemical recurrence, then in the initial staging of intermediate and high-risk disease, and finally in the evaluation of advanced disease. In addition, randomized trials with new hormonal agents are being designed with innovative primary endpoints based on PSMA PET/CT findings^(27)^. In the near future, it may be imperative that new imaging modalities become part of public health practice. Given the high cost of PSMA PET/CT, there is a need for feasibility studies regarding its adoption in Brazil.

This first experience of performing PSMA PET/CT in patients with biochemical recurrence of prostate cancer treated via the SUS revealed at least three important pieces of information. First, the information provided by this imaging method had a significant impact on therapeutic management, as has been observed worldwide^(21)^. Because of that information, the treatment strategy was altered in more than half (61.0%) of the patients in our sample, which was similar to the (56.8%) found in the multicenter IAEA study^(21)^. Overall, this impact was mainly observed as a shift away from salvage therapy and toward systemic therapy, which occurred in 18 (30.5%) of the 59 cases analyzed. Because this represents choosing not to administer a potentially curative treatment, it seems that, for these patients, PSMA PET/CT revealed the extent of the disease to be greater than what had been thought on the basis of conventional imaging findings and clinical data. Hence, in these particular cases, the information provided by PSMA PET/CT made it possible to avoid treatments with curative intent which would actually have been futile and poorly indicated, including five surgical procedures (two prostatectomies and three lymphadenectomies). Those treatments were ultimately replaced with systemic therapy. Second, adding PSMA PET/CT to the workflow resulted in higher costs. This was expected, given that the technology is still quite expensive, relying on imported supplies and demanding high-cost facilities and personnel. In contrast to PSMA PET/CT-related costs found in the medical literature^(28)^, its cost in Brazil ($524.19) seems to be lower than in high-income countries such as Switzerland ($2,737.88), Israel ($1,801.85), Denmark ($1,387.90), and Australia ($680.27). This could be explained by the overall lower supply costs in a country with lower comparative incomes such as Brazil, although we refrain from further speculation in our analysis. Third, and perhaps more interestingly, the higher cost observed for the after-PSMA PET/CT strategy cannot be solely explained by the costs of performing the examination. In fact, the PSMA PET/CT imaging costs ($524.19) are equivalent to only approximately half of the cost of not performing it, given that the mean cost difference between the two strategies in the three scenarios was $1,028.32. This implies that the other half of these cost differences (between performing and not performing PSMA PET/CT) comes from the shift in the therapeutic management itself. We demonstrated a trend toward a shift from salvage to systemic therapy resulting from knowledge of the PSMA PET/CT findings. We can assume that lowering the rate of salvage treatment and increasing that of systemic treatment entails higher costs, as it did in the time window evaluated in our study, albeit narrow (24 months).

In our study sample, the GS at prostate biopsy, mean PSA level, and mean PSA doubling time were comparable to the overall distribution of those reported in the IAEA study^(21)^: Our findings, in comparison with those of that study, were as follows: GS7, 66.1% vs. 61.1%; GS8, 16.9% vs.19.5%; GS9, 15.2% vs. 17.9%; GS10, 1.7% vs. 1.5%; mean PSA level at the time of PSMA PET/CT, 1.7 ng/mL, the same as in the IAEA study; and mean PSA doubling time, 11.2 months vs. 11.18 months.

It should be borne in mind that this change in the treatment strategy actually represents avoiding curative therapies that would have been futile and were ultimately not performed because PSMA PET/CT allowed a better understanding of the real disease burden. Therefore, another hypothesis comes to mind: perhaps, from our narrow time window perspective, these higher costs in the after-PSMA PET/CT strategy actually represent an anticipation of expenditures. The reasoning behind this is that these higher costs presented themselves only after futile curative treatments were avoided, and to a great extent (in 35.6% of cases). To better illustrate this point, these correspond to the initial salvage treatment strategies for 21 patients, which were radical prostatectomy in two, pelvic lymphadenectomy in three, and radiotherapy alone in 16. The total amount saved by not providing those treatments over the 24-month study period was $22,562.20, which would have been expended inappropriately. If those funds were directed towards PSMA PET/CT scans (unit cost, $524.19), they would suffice for evaluating 43 patients (roughly two patients for each futile treatment avoided). Therefore, we highlight the need for similar studies with broader time windows, hypothesizing that, in the long run, the economic savings of not performing PSMA PET/CT early in the workflow might be outweighed in the future by the costs of performing futile curative treatments and later also by the costs of the systemic therapies to which these patients would end up being submitted to anyway.

Although we tried to approach the costs involved in each component of the study in great detail, the impossibility of actually gathering every conceivable cost should be highlighted as a limitation of our study, especially regarding costs related to complications of the treatments (and, to a lesser extent, of the imaging methods). Unfortunately this information was not available in a consistent manner. Another limitation, as previously mentioned, was our narrow time window, which did not allow for more robust ascertainments regarding the cost differences. A third limitation pertains to the lack of quality of life assessment metrics. That analysis could not be performed, mainly due to constraints imposed by the data collection method: because the study was based on the IAEA multicenter study forms^(21)^, which also did not assess this topic, it could not be properly evaluated. We highlight the importance of obtaining such information for future cost effectiveness studies, in which quality of life assessments are paramount.

## CONCLUSION

For patients treated via the SUS, the use of PSMA PET/CT incurs higher costs than does the use of conventional imaging methods. Adding PSMA PET/CT to the workflow seems to have an impact on the therapeutic management of biochemical recurrence of prostate cancer, mainly promoting a shift from futile curative treatments to systemic palliative ones. The amount of funds that could potentially be redirected away from futile treatments would suffice to evaluate roughly two patients with PSMA PET/CT scans for every futile treatment avoided.
